# Lichen planopilaris versus frontal fibrosing alopecia: histopathologically distinct diseases or not?^؆^

**DOI:** 10.1016/j.abd.2026.501302

**Published:** 2026-03-23

**Authors:** Deren Özcan, Deniz Seçkin, A.T. Güleç, Özlem Özen

**Affiliations:** a Department of Dermatology, Faculty of Medicine, Başkent University, Ankara, Turkey; b Department of Pathology, Faculty of Medicine, Başkent University, Ankara, Turkey

Dear Editor,

Lichen Planopilaris (LPP) and Frontal Fibrosing Alopecia (FFA) are primary lymphocytic cicatricial alopecia types.^1^, ^2^ Because of their similar histopathology, namely perifollicular lichenoid inflammation and concentric fibrosis involving the infundibulum and isthmus, most authors consider FFA as a clinical variant of LPP.^1^, ^2^, ^3^, ^4^ However, FFA has a unique clinical pattern with progressive frontotemporal hairline recession accompanied by eyebrow loss, whereas LPP classically presents with patchy alopecia over the central scalp.^1^, ^4^ Additionally, hormonal factors in the context of postmenopausal predominance, neurogenic inflammation and environmental triggers like sunscreen or leave-on facial products may contribute to FFA etiopathogenesis.^3^ Treatments for FFA and LPP also differ in certain aspects; finasteride and dutasteride are the most beneficial agents for the former, while they do not work for the latter.^1^, ^3^ Therefore, it is questionable whether FFA can be considered a form of LPP based solely on histopathologic resemblance. Only a few studies compared the histopathology of LPP and FFA. However, they either included a small number of specimens or evaluated only transverse sections.^1^, ^5^, ^6^, ^7^, ^8^ We compared the histopathologic findings of LPP and FFA in a considerable number of patients by examining both transverse and vertical sections to determine if these diseases can be regarded in the same spectrum or represent distinct entities.

The study was approved by the local Institutional Review Board.

42 cases with LPP and 19 cases with FFA, diagnosed based on classical clinical and histopathologic findings^1^, ^2^, ^3^, ^4^ between March 2006 and January 2017, were included. The hair pathologist, blinded to the diagnoses, re-evaluated the transverse and vertical sections of two 4-mm scalp punch biopsy specimens and recorded the findings to the checklist recommended by the North American Hair Research Society.^9^ Additional findings and the localization of inflammation were also noted. We compared the frequency of each finding between LPP and FFA, and statistically analyzed the differences.

All patients with FFA were females (mean age, 55.6-years). The LPP group included 27 females and 15 males (mean age, 47.4-years). Histopathologically, perifollicular lichenoid/interface dermatitis and perifollicular fibrosis were noted in all patients. Fig. 1, Fig. 2 demonstrates the notable histopathologic findings in LPP and FFA; Table 1 shows the comparison of the histopathologic findings in each group.

**Fig. 1:**
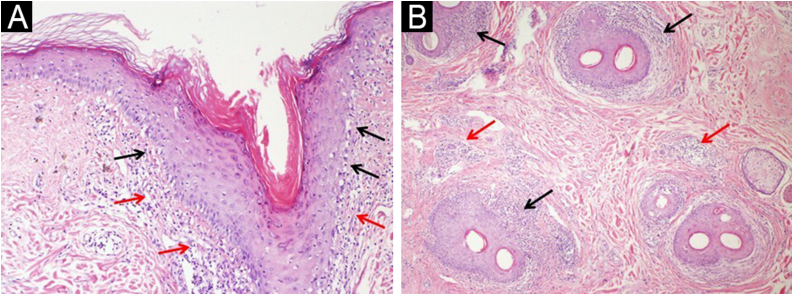
Lichen planopilaris. (A) Vacuolar degeneration of infundibulum basal cells (black arrows) and dermal lichenoid infiltrate (red arrows) (vertical section, Hematoxylin & eosin, ×200). (B) Perifollicular lichenoid inflammation (black arrows) and interfollicular interstitial/perivascular inflammation (red arrows) (transverse section, Hematoxylin & eosin, ×100).

**Fig. 2:**
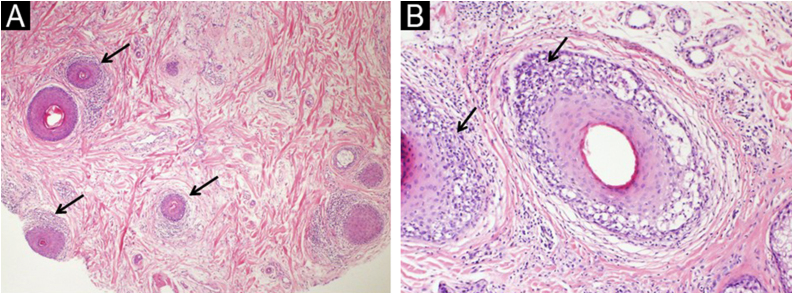
Frontal fibrosing alopecia. (A) Follicular miniaturization and perifollicular lichenoid inflammation of miniaturized follicles (arrows) (transverse section, Hematoxylin & eosin, ×100). (B) Follicular lichenoid inflammatory infiltration (arrows) (transverse section, Hematoxylin & eosin, ×100).

**Table 1:** Comparison of the histopathologic findings in 42 cases with LPP and 19 cases with FFA.

	LPP	FFA	
Histopathologic findings^a^	N of cases (%)	N of cases (%)	p-value^b^
***Epidermal changes***	34 (81)	10 (52.6)	**0.032**
**Lichenoid inflammatory infiltration**	14 (33.3)	0 (0)	**0.003**
**Vacuolar degeneration of basal cells**	12 (28.6)	1 (5.3)	**0.047**
Spongiosis	6 (14.3)	1 (5.3)	0.418
Atrophy	5 (11.9)	4 (21.1)	0.441
Acanthosis	13 (31)	4 (21.1)	0.544
*Severity of inflammation*			
Mild	16 (38.1)	9 (47.4)	0.166
Moderate	19 (45.2)	10 (52.6)	0.160
Severe	7 (16.7)	0 (0)	0.166
*Localization of inflammation*			
**Interfollicular interstitial**	12 (28.6)	0 (0)	**0.012**
Interfollicular perisudoriparous gland	40 (95.2)	19 (100)	1
Interfollicular perivascular	35 (83.3)	16 (84.2)	1
Subcutaneous	42 (100)	19 (100)	‒
Follicular infundibulum	16 (38.1)	8 (42.1)	0.784
Follicular isthmus	40 (95.2)	17 (89.5)	0.582
Peribulbar	5 (11.9)	0 (0)	0.313
Follicular dermoepithelial junction	40 (95.2)	19 (100)	1
Papillary dermis	31 (73.8)	14 (73.7)	1
Reticular dermis	28 (66.7)	13 (68.4)	1
***Miniaturization of terminal follicles***^c^	13 (31)	11 (57.9)	**0.044**
*Terminal hair density*			0.920
Normal/mild reduction	4 (9.5)	2 (10.5)	
Moderate reduction	32 (76.2)	15 (78.9)	
Marked reduction/absent	6 (14.3)	2 (10.5)	
*Vellus hair density*^c^			0.543
Normal	30 (73.4)	13 (68.4)	
Increased	3 (7.1)	3 (15.8)	
Absent	9 (21.4)	3 (15.8)	
*Follicular unit structure*			0.162
Normal	22 (52.4)	14 (73.7)	
Destructed	20 (47.6)	5 (26.3)	
*Sebaceous glands of involved follicles*			0.219
Normal	4 (9.5)	3 (15.8)	
Atrophic	17 (40.5)	11 (57.9)	
Total loss	21 (50)	5 (26.3)	
*Follicular changes*			
Lichenoid inflammatory infiltration	40 (95.2)	17 (89.5)	0.582
Vacuolar degeneration of basal cells	2 (4.8)	2 (10.5)	0.582
Spongiosis	1 (2.4)	1 (5.3)	0.530
Politrichia	9 (21.4)	3 (15.8)	0.737
Lymphocytic exocytosis	37 (88.1)	16 (84.2)	0.695
Dilatation	10 (23.8)	4 (21.1)	1
Naked hair shafts	10 (23.8)	5 (26.3)	1
Single cell necrosis of keratinocytes	19 (45.2)	8 (42.1)	1
Apoptosis of the follicular sheath	6 (14.3)	3 (15.8)	1
Abnormal inner root sheath desquamation	3 (7.1)	0 (0)	0.545
Atrophy/necrosis	2 (4.8)	0 (0)	1
Follicular destruction			
Focal	18 (42.9)	10 (52.6)	0.582
Complete	8 (19)	1 (5.3)	0.251
*Perifollicular fibrosis*			
Above bulge			0.201
Absent	10 (23.8)	3 (15.8)	
Concentric lamellar fibroplasia	18 (42.9)	13 (68.4)	
Mucinous fibroplasia	13 (31)	2 (10.5)	
Hyalinization	1 (2.4)	1 (5.3)	
Below bulge			0.198
Absent	20 (47.6)	8 (42.1)	
Concentric lamellar fibroplasia	9 (21.4)	8 (42.1)	
Mucinous fibroplasia	13 (31)	3 (15.8)	
Hyalinization	0 (0)	0 (0)	
*Follicular tract*			
Absent	1 (2.4)	3 (15.8)	0.085
Fibrovascular	20 (47.6)	10 (52.6)	0.786
Hyalinized	17 (40.5)	7 (36.8)	1
Mucinous/elastotic fibroplasia	8 (19)	3 (15.8)	1
*Elastic fiber pattern*			0.407
Normal	27 (64.3)	13 (68.4)	
Perifollicular scar	14 (33.3)	5 (26.3)	
Superficial perifollicular wedge-shaped scar	0 (0)	1 (5.3)	
Diffuse scar (involves interfollicular dermis)	1 (2.4)	0 (0)	

FFA, Frontal Fibrosing Alopecia; LPP, Lichen Planopilaris; n, Number.

a To prepare transverse sections, biopsy samples were divided transversely into three or four equal pieces, approximately 1 mm thick, to obtain samples from different skin levels. Histopathologic findings with statistically significant differences are highlighted in bold.

b The difference in the frequency of each finding between LPP and FFA was statistically analyzed using χ² and Fisher’s exact tests; p-values less than 0.05 were considered significant and are highlighted in bold.

c Follicular miniaturization was identified by follicular bulbs present in the mid-to-deep dermis, hair shafts thinner than their inner root sheath and shafts narrower than 0.06 mm. Vellus hairs were identified by also having thin shafts (≤0.03 mm), but with bulbs located in the upper portion of the dermis.

Although some histopathologic differences between LPP and FFA have been shown, these are considered subtle or non-specific to reliably distinguish the two conditions.^1^, ^5^, ^6^, ^7^, ^8^ Reportedly, FFA exhibits more pronounced apoptosis and less but deeper inflammation compared to LPP.^1^, ^7^, ^8^ Besides, interfollicular epidermis tends to be affected more and concentric lamellar fibroplasia is severe in LPP.^1^, ^5^, ^7^ Herein, the frequency of epidermal changes, specifically lichenoid inflammation and vacuolar alterations, was significantly higher in LPP compared to FFA. Another notable finding was the presence of interfollicular interstitial lymphocytic infiltration exclusively in LPP cases. These findings suggest that LPP is characterized by a lymphocytic infiltrate which involves the whole epidermis and dermis, while FFA is characterized by a more follicle-centered lymphocytic inflammation. Although severe inflammation and deeper follicular level involvement were more common in LPP, the difference between the two alopecias was not significant. The higher frequency of miniaturization in FFA was likely due to concomitant androgenetic alopecia or fibrosing alopecia in a pattern distribution.

The exact mechanisms underlying LPP and FFA remain unclear, but involve irreversible loss of stem cells in the hair follicle bulge that sustain follicle cycling and repair.^2^, ^4^ Therefore, any stimulus that collapses bulge immune privilege may predispose follicular stem cells to T-cell-dependent cytotoxic damage, causing lichenoid inflammation and permanent alopecia in immunogenetically susceptible individuals.^2^, ^3^ A lichenoid reaction can be precipitated by various factors, such as mechanical trauma, contact sensitivity, and some viruses, and can also be observed in dermatoses like drug reactions and graft-versus-host disease.^10^ Thus, there appears to be a similar reaction pattern triggered by diverse stimuli, albeit targeting the bulge region of terminal follicles in LPP and androgen-dependent hair follicles of the frontal scalp in FFA. It is likely that diffuse LPP variants also represent phenotypically distinct branches of the same pathogenetic process.

We propose that LPP and FFA may represent distinct diseases that exhibit a similar pattern of lymphocyte-mediated follicular reaction, albeit with a different extent of epidermal and interfollicular dermal involvement. Further research could provide deeper insights into the relationship between these alopecias and aid in developing targeted therapies.

## ORCID ID

Deren Özcan: 0000-0002-7450-6886

Deniz Seçkin: 0000-0003-1913-9734

A. Tülin Güleç: 0000-0003-0876-5561

Özlem Özen: 0000-0002-9082-1317

## Financial support

This research did not receive any specific grant from funding agencies in the public, commercial, or not-for-profit sectors.

## Author’s contributions

Deren Özcan: Approval of the final version of the manuscript; critical literature review; data collection, analysis and interpretation; effective participation in research orientation; intellectual participation in propaedeutic and/or therapeutic management of studied cases; manuscript critical review; preparation and writing of the manuscript; statistical analysis; study conception and planning.

Deniz Seçkin: Approval of the final version of the manuscript; critical literature review; data collection, analysis and interpretation; participation in propaedeutic and/or therapeutic management of studied cases; manuscript critical review; study conception and planning.

A. Tülin Güleç: Approval of the final version of the manuscript; critical literature review; data collection, analysis and interpretation; participation in propaedeutic and/or therapeutic management of studied cases; manuscript critical review; study conception and planning.

Özlem Özen: Approval of the final version of the manuscript; data collection, analysis and interpretation; effective participation in research orientation; manuscript critical review; study conception and planning.

## Research data availability

Does not apply.

## Conflicts of interest

None declared.
